# *In vivo* parcellation of the human spinal cord functional architecture

**DOI:** 10.1162/imag_a_00059

**Published:** 2024-01-11

**Authors:** Nawal Kinany, Caroline Landelle, Benjamin De Leener, Ovidiu Lungu, Julien Doyon, Dimitri Van De Ville

**Affiliations:** Department of Radiology and Medical Informatics, University of Geneva, Geneva, Switzerland; Neuro-X Institute, Ecole Polytechnique Fédérale de Lausanne (EPFL), Geneva, Switzerland; McConnell Brain Imaging Centre, Department of Neurology and Neurosurgery, Montreal Neurological Institute, McGill University, Montreal, Canada; Department of Computer Engineering and Software Engineering, Polytechnique Montreal, Montreal, Canada; CHU Sainte-Justine Research Centre, Montreal, Canada; Département de psychiatrie et addictologie, Université de Montréal, Montréal, Canada

**Keywords:** fMRI, spinal cord, functional connectivity, data-driven, resting-state networks

## Abstract

The spinal cord is a critical component of the central nervous system, transmitting and integrating signals between the brain and the periphery *via* topographically organized functional levels. Despite its central role in sensorimotor processes and several neuromotor disorders, mapping the functional organization of the spinal cord *in vivo* in humans has been a long-standing challenge. Here, we test the efficacy of two data-driven connectivity approaches to produce a reliable and temporally stable functional parcellation of the cervical spinal cord through resting-state networks in two different functional magnetic resonance imaging (fMRI) datasets. Our results demonstrate robust and replicable patterns across methods and datasets, effectively capturing the spinal functional levels. Furthermore, we present the first evidence of spinal resting-state networks organized in functional levels in individual participants, unveiling personalized maps of the spinal functional organization. These findings underscore the potential of non-invasive, data-driven approaches to reliably outline the spinal cord’s functional architecture. The implications are far-reaching, from spinal cord fMRI processing to personalized investigations of healthy and impaired spinal cord function.

## 1 Introduction

The spinal cord is an essential component of the central nervous system (CNS) acting as a pivotal neural relay and processing center at the core of the sensorimotor hierarchy (Bican et al., 2013). Its circuitry is topographically organized and comprises distinct pools of motor and sensory neurons that, in collaboration with interneurons, transmit and integrate signals between the brain and periphery through spinal nerve roots (Pierrot-Deseilligny & Burke, 2012). These pools of neurons are distributed throughout the spinal cord in distinct levels, which form functional units of the spinal architecture and give rise to pairs of nerves emerging between the vertebrae to connect to specific body parts.

Despite its central importance in human sensorimotor processes and its involvement in various neurological conditions such as spinal cord injuries (Ahuja et al., 2017), multiple sclerosis (Filippi et al., 2018), cervical myelopathy (Nouri et al., 2022), and chronic pain (Cheng, 2010), the large-scale spinal cord’s functional organization remains largely unexplored *in vivo* in humans. Instead, traditional methods have primarily focused on characterizing its structure, relying on cadaver dissection (Kameyama et al., 1996; Ko et al., 2004; Mendez et al., 2021; Panjabi et al., 1991) or structural magnetic resonance imaging (MRI) (Cadotte et al., 2015) to identify vertebral bones (referred to as *vertebral* levels) or nerve roots (known as *segmental* levels). While these approaches have provided valuable insights into the spinal cord’s anatomy, they fall short in capturing the rich neuronal circuitry that underlies its functions (*i.e.*, its *functional* levels). Therefore, it is crucial to advance reliable *in vivo* methods to map the functional architecture of the spinal cord. Such developments are essential to achieve a comprehensive understanding of its organization in healthy individuals and to illuminate the alterations it undergoes in various neurological conditions.

In fact, elucidating the functional architecture of the CNS has been a long-standing challenge in neuroscience. Over the past decades, functional magnetic resonance imaging (fMRI) has emerged as a powerful tool for mapping neural activity (Power et al., 2014) under multiple conditions. Among them, resting-state recordings stand out for their minimal experimental burden and have proven particularly useful for unraveling neural circuits, enabling researchers to study a wide range of participants, including clinical populations (Lee et al., 2013). Assessing the brain’s spontaneous activity has provided valuable information about its intrinsic organization, notably by using a variety of analytical approaches to delineate patterns of coordinated activity, the so-called resting-state networks (Biswal et al., 2010; Fox & Raichle, 2007). They have demonstrated a considerable degree of consistency across methods and datasets (Chen et al., 2008; Damoiseaux et al., 2006), have yielded insights into individual-specific neural organization (Finn et al., 2015; Gordon, Laumann, Adeyemo, et al., 2017; Wang et al., 2015), and have proven relevant to both normal and pathological brain function (Hohenfeld et al., 2018).

Yet, while fMRI has been extensively deployed to disentangle brain activity, its application to the spinal cord has faced significant challenges (*e.g.,* size, inhomogeneous magnetic field, physiological noise) that have slowed progress in this area. Although recent years have witnessed an emergence of studies leveraging technological advances to probe spinal cord activity, explorations of spontaneous activity of the spinal cord at rest remain scarce (for reviews, see Harrison et al. (2021), Kinany, Pirondini, Micera, et al. (2022), Landelle et al., (2021)). Nonetheless, a handful of studies uncovered spinal cord resting-state networks, hence confirming that organized spontaneous fluctuations are, indeed, a ubiquitous feature of the CNS. These studies notably utilized data-driven techniques, such as independent component analysis (ICA; Kong et al., 2014; Landelle et al., 2023; Vahdat et al., 2020) or innovation-driven co-activation pattern analysis (iCAP; Kinany, Pirondini, Mattera, et al., 2022; Kinany et al., 2020, 2023), to reveal spatially segregated patterns of activity at rest. These two methods extract functional networks using distinct approaches: ICA aims to retrieve spatially independent components by maximizing a measure of their statistical independence (Beckmann & Smith, 2004; Calhoun et al., 2001; Varoquaux et al., 2010). While spatial ICA has dominated the application to fMRI and is employed in this study, it is worth noting that temporal ICA can also be used to map temporally independent resting-state networks (Smith et al., 2012). On the other hand, iCAP is a dynamic functional connectivity method that focuses on identifying components characterized by similar temporal dynamics (Karahanoğlu & Van De Ville, 2015). To achieve this, it relies on the temporal clustering of individual frames of transient activity and explicitly accounts for temporal blurring caused by the hemodynamic response function.

Here, we argue that the investigation of spinal resting-state networks presents an untapped potential to disentangle the intricate functional architecture of the spinal cord *in vivo* and, in particular, its functional levels. Although previous research has highlighted the organized nature of spinal resting-state networks, no study has systematically characterized their capability to reliably map the topographic organization of these functional units. To address these gaps, we leveraged and compared two techniques for network extraction, ICA and iCAP, across two distinct cervical spinal cord fMRI datasets; one collected in Montreal (“mtl”) and the other in Geneva (“gva”). Our study had three primary objectives: firstly, to evaluate the efficacy of these methods in achieving a functional mapping of spinal cord levels; secondly, to systematically assess the reliability and generalizability of this mapping by probing its robustness (*i.e.,* across methods), replicability (*i.e.,* across datasets), and stability (*i.e.,* across time); and finally, to present novel evidence of spinal resting-state networks at the individual level, showcasing their potential to uncover personalized maps of spinal functional levels.

Altogether, our findings provide unprecedented support for the potential of spinal cord resting-state networks as reliable indicators of the spinal cord’s functional organization, at the group and individual scales. This study holds great promise for advancing our understanding of human sensorimotor circuits and unlocking new insights into residual neural function and nerve root damage in individuals with spinal cord injuries or radiculopathies. Mapping the spinal cord’s functional architecture using spinal cord fMRI and data-driven functional connectivity approaches may have significant translational implications, paving the way to custom clinical assessments, pre-surgical mapping, as well as treatment planning and monitoring.

## 2 Methods

### 2.1 Participants & acquisition

Two T2*-weighted gradient-echo echo-planar imaging datasets of healthy participants were used in this study, namely the “gva” dataset (data acquired at Campus Biotech Geneva (Kinany et al., 2020)) and the “mtl” dataset (data acquired at the Neuro, Montreal Neurological Institute (Landelle et al., 2023)). Both datasets were acquired using a 3-Tesla MRI Scanner (Magnetom-Prisma, Siemens, Erlangen, Germany) equipped with a 64-channel head (“gva”: inferior element 7 active, “mtl 1-7 elements active) and neck coil (1-2 elements active). In both datasets, functional data were acquired during rest (*i.e.,* no explicit task) with eyes open. Participants were instructed to relax and to minimize motion and swallowing. Physiological recordings were acquired using a pulse sensor and a respiration belt (“gva”: Biopac MP150 system, California, USA, “mtl”: Siemens Physiology Monitoring Unit).

#### 2.1.1 Dataset “gva”

Nineteen right-handed healthy participants (11 females, 28.5 ± 3.5 years old) from the “gva” dataset were included in the study. All participants gave their written informed consent in accordance with the Helsinki Declaration, and the study was approved by the Commission Cantonale d’Éthique de la Recherche Genève (CCER, study 2019-00203). Functional images (*i.e.,* blood-oxygen-level-dependent – BOLD – time series) were acquired using a ZOOMit selective field-of-view imaging, focusing on the cervical enlargement (Repetition Time (TR) = 2500 ms, Echo Time (TE) = 34 ms, axial field of view (FOV) = 48 x 144 mm^2^, flip angle = 80°, in-plane resolution = 1 x 1 mm^2^, slice thickness = 3 mm, transversal acquisition, number of slices = 32, number of volumes = 360; duration = 15 min). High-resolution anatomical images covering a region from C1 to the upper part of the thoracic spine were acquired using a T2-weighted image (TR/TE = 1500/135 ms, echo train length = 74, flip angle = 140°, resolution = 0.4 x 0.4 x 0.8 mm^3^, sagittal acquisition).

#### 2.1.2 Dataset “mtl”

Twenty-one right-handed healthy participants (16 females; age 50.1 ± 14.1 years old) from the “mtl” dataset were included in this study. We included the 12 younger healthy controls (age under 65 years old) from our previous study (Landelle et al., 2023) and introduced an additional 9 new participants. The experiment was approved by the local ethics committee (MUCH REB 2019-4626), and all participants gave their written consent in accordance with the Helsinki Declaration. Functional images were acquired using a multiband gradient-echo EPI sequence covering the brain and cervical spinal cord (TR/TE = 1550/23 ms, axial FOV = 192 x 192 mm^2^, generalized autocalibrating partially parallel acquisition (GRAPPA) with integrated parallel acquisition technique (iPAT) acceleration factor for phase encoding direction = 2 and multiband factor for slice encoding direction = 3, flip angle = 70°, in-plane resolution = 1.6 x 1.6 mm^2^, slice thickness = 4 mm, transversal acquisition, number of slices = 69, number of volumes = 230, duration = 6 min). It is worth noting that multiband acquisitions may be susceptible to slice leakage artifacts (Risk et al., 2021; Todd et al., 2016). While the chosen values are expected to mitigate this issue, it is important to acknowledge that a systematic analysis of its impact has not been conducted in this study. Anatomical images were acquired using a high-resolution T1-weighted sequence covering the whole brain and the cervical spinal cord up to T1 vertebrae in most participants (TR/TE = 2300/3.3 ms, MPRAGE sequence, GRAPPA iPAT acceleration factor = 2, flip angle = 9°, resolution = 1.3 x 1.3 x 1.3 mm^3^, transversal acquisition). A T2*-weighted sequence covering the whole cervical spinal cord was also acquired from C1 to C7 vertebrae in most participants (TR/TE = 34/14 ms, flip angle = 5°, resolution = 0.47 x 0.47 x 5 mm^3^, transversal acquisition). For both functional and anatomical scans, only slices covering the spinal cord were kept for the following analyses.

### 2.2 Data processing

The spinal cord functional and structural images were pre-processed using an in-house pipeline (for details see Landelle et al. (2023)) based on the Spinal Cord Toolbox (SCT, version 5.0) (De Leener et al., 2017), the Oxford Center for fMRI of the Software Library (FSL, version 5.0), the Tapas PhysiO toolbox (release 2022a, V8.1.0) (Kasper et al., 2017), and the Nilearn toolbox (version 0.9.1), a Python package that uses scikit-learn library (Abraham et al., 2014). The same pipeline was applied to both datasets, except for the type of anatomical image used, which was T2w for “gva” and T1w for “mtl.” Signal quality (motion and temporal signal-to-noise ratio) were evaluated in both datasets (see Supplementary information and Figure S1).

#### 2.2.1 Preprocessing

The following preprocessing steps were performed: i) slice-timing correction (FSL), ii) motion correction using slice-wise realignment and spline interpolation (with SCT, sct_fmri_moco). Outlier volumes were identified within the spinal cord mask for subsequent motion scrubbing (with FSL, using the root mean square intensity difference of volume N to volume N+1, *i.e.*, DVARS), iii) segmentation of functional and structural images (with SCT, sct_deepseg, followed my manual correction), iv) time series denoising (see details in Time series denoising), v) coregistration of functional images to anatomical image and, then, to the PAM50 template (with SCT, sct_register_multimodal and sct_register_to_template), and vi) anisotropic smoothing using a 3D Gaussian kernel with a full width half maximum (FWHM) of 2 x 2 x 6 mm^3^ for “gva” and 3 x 3 x 6 mm^3^ for “mtl” (with nilearn, nilearn.image.smooth_img).

#### 2.2.2 Time series denoising

For each participant, we modeled nuisance regressors to account for physiological noise using the Tapas PhysiO toolbox (Kasper et al., 2017). First, we used the CompCor approach (Behzadi et al., 2007) to identify non-neural fluctuations by extracting the first five principal components of the unsmoothed cerebrospinal fluid (CSF) signal in the participant’s native space. Second, we generated noise regressors from peripheral physiological recordings (heart rate and respiration) using the RETROspective Image CORrection (RETROICOR) procedure (Glover et al., 2000). Specifically, we modeled four respiratory, three cardiac harmonics, and one multiplicative term for the interactions between respiratory and cardiac noise (18 regressors in total, similar to Harvey et al. (2008); Kinany et al. (2019); Tinnermann et al. (2017)). The first five discrete cosine transform (DCT) basis functions were added for detrending. These nuisance regressors were combined with the two motion parameters (x and y) and motion outliers. The removal of the noise confounds was based on a projection on the orthogonal of the fMRI time-series space and was applied orthogonally to the high-pass temporal filter (0.01 Hz) using the Nilearn toolbox (*clean_img* function). This noise modeling approach has the advantage of preventing the reintroduction of artifacts that had previously been removed from the data (Lindquist et al., 2019).

### 2.3 Extraction of resting-state networks

Two data-driven approaches (ICA and iCAP) were deployed to identify spinal cord resting-state networks in each dataset. Components were extracted at the group-level as well as for each individual participant.

#### 2.3.1 ICA approach

Generalized canonical correlation analysis (CanICA or ICA) is a hierarchical model for group-level ICA analysis that has demonstrated a high degree of component reproducibility (Varoquaux et al., 2010). The estimation procedure is based on three main steps: i) The 20 principal components that explained most of the variance for a given participant were selected at the participant level using a principal component analysis. This procedure guaranteed uniformity among participants, ensuring that each individual had an identical number of components utilized for the selection of independent components at the group level. These patterns of interest were then concatenated across participants. ii) A canonical correlation analysis was used to identify the subspace common to the group. iii) Source separation was performed using spatial ICA on the previous group-subspace (500 iterations) in order to retrieve spatially independent components at group level. Note that for analysis conducted at the participant level only the last step was applied.

#### 2.3.2 iCAP approach

By contrast, the iCAP framework (Karahanoğlu & Van De Ville, 2015; Kinany et al., 2020) employs hemodynamic deconvolution to retrieve temporal dynamics of spatially overlapping networks identified by similar transient activities. Using denoised time series, activity-inducing signals were extracted by applying Total Activation, a regularized deconvolution of fMRI signals (Karahanoğlu et al., 2013). Transients, also referred to as innovation signals, were obtained by calculating the temporal derivative of these activity-inducing time courses. A two-step thresholding process was then used to select significant innovation frames: i) temporal thresholding based on a 5% confidence interval derived from a surrogate distribution generated by applying TA to phase-randomized data, and ii) spatial thresholding to retain only frames containing more than 5% active voxels. Frames with significant and similar transitioning activities were k-means clustered together to obtain innovation-driven coactivation patterns (iCAPs).

#### 2.3.3 Number of components

**Fig. 1.**
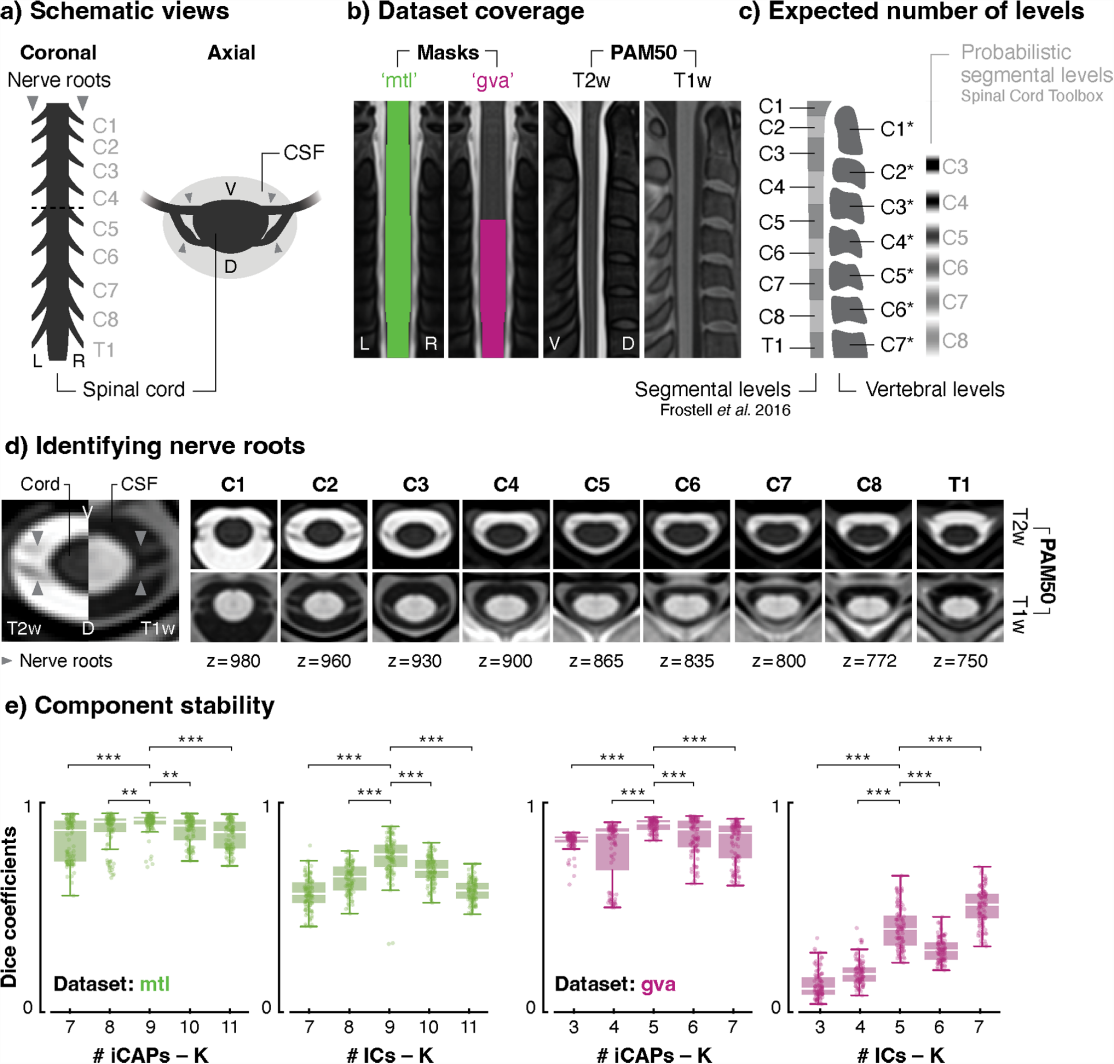
Number of spinal levels and components. (a) Schematic views of the upper spinal cord depicting pairs of nerves (formed by dorsal and ventral nerve roots), corresponding to spinal segmental levels C1 to T1. Dashed line represents the axial view. (b) Masks coverage of the functional analyses displayed on coronal views of the PAM50-T2w template. Sagittal views of the PAM50-T1w and -T2w are provided to identify vertebrae. (c) Schematic views of spinal segmental and vertebral (indicated with a *) levels. The expected number of segmental levels in each dataset was determined using anatomical tables (Frostell et al., 2016) and hypothesized to match the number of functional levels. The analysis masks encompass nine segmental levels for “mtl” (*i.e.*, levels C1 to T1, facing vertebrae C1* to C7*) and five for “gva” (*i.e.*, levels C5 to T1, facing vertebrae C4* to C7*). Probabilistic segmental levels from the SCT are provided as reference (De Leener et al., 2017), emphasizing the distinction between these conventionally used levels and those utilized in the current study. Of note, from SCT v6.1, the atlas of segmental levels has been modified to use levels reported in Frostell et al. (2016). (d) We confirmed the expected number of spinal segmental levels by visually identifying nerve roots in PAM50 T2w and T1w images. The left panel indicates the structures of interest, while the right panels show representative slices for each spinal segmental level in both imaging modalities. Coordinates are in PAM50 space. (e) We tested the stability of the selected number of components for both datasets, by extracting components in random subsets of the data (10 participants in 100 repetitions, bootstrap across participants with replacement) for each targeted K (*i.e.*, 9 for “mtl” and 5 for “gva”) ± 2. The similarity of these components with those extracted in the full datasets was evaluated. Each box represents the distribution (*i.e.,* from the 25th to the 75th percentile) of Dice coefficients for each K value, with medians represented by the horizontal white line inside the box. Vertical lines denote the extreme values within a 1.5 interquartile range and dots represent Dice values for each of the 100 subsets. CSF: cerebrospinal fluid, L: left, R: right, V: ventral, D: dorsal. ** p < 0.01, *** p < 0.001, FDR-corrected.

We hypothesized that the number of spinal *functional* levels (*i.e.*, the spinal networks extracted using data-driven connectivity approaches) should align with the number of spinal *segmental* levels (*i.e.,* anatomically identified nerve roots). This hypothesis is driven by the goal of isolating topographically organized signals associated with the neuron pools giving rise to each pair of nerve roots. Thus, we aimed to match the number of components extracted with ICA and ICAP analyses (denoted as K), with the expected number of spinal segmental levels. To account for the different coverages of each dataset, K was independently defined for “mtl” and “gva.” The expected number of spinal cord segmental levels was determined using anatomical knowledge (Frostell et al., 2016, SCT v6.1 or later) and was further verified through visual identification of nerve roots in the PAM50 template space (Fig. 1). Note that we opted not to use the probabilistic levels from the SCT (De Leener et al., 2017), and favored instead measurements summarized from Frostell et al. (2016), which aggregated data from multiple studies and more closely aligned with the nerve roots. Levels of the SCT are provided for reference. Respectively, five and nine spinal cord levels were expected for “gva” and “mtl” datasets. To validate the choice of K values, we implemented a subsampling scheme that systematically evaluated the similarity of components extracted from random subsets of the dataset to those extracted from the entire dataset. We generated 100 subsets of 10 participants for each dataset (bootstrap across participants with replacement) and extracted iCAPs and ICs for the targeted K ± 2 components (*i.e.*, “gva”: 5 ± 2 and “mtl”: 9 ± 2 components). In both ICA and iCAP approaches, the resulting component maps appear as blobs of voxels with higher intensity standing out from a background voxel intensity. Thus, the voxel intensity was normalized using a Z-score, and a threshold of Z > 1.6 was applied to retain only the tail of the voxel intensity distribution (~5% of the map’s voxels). Resulting component maps were binarized and the stability of the components across the subsets was established using Dice coefficients between subset maps and whole-group maps (computed as the ratio between twice the number of overlapping voxels and the sum of the number of voxels in the two maps). Dice coefficients were statistically tested between each K using a linear mixed model (*lmer* function in the R package “lme4”) which took into account the effect of K values (fixed-effect), the variability between “subsets” (random effects) and the residual error. Main effect was analyzed using Wald test (*Anova* function in the R package “car”), and effect sizes were calculated using Cohen’s d coefficients (d, *chisq_to_cohens_w* function in the R package “effectsize”). Significant main effects (p < 0.05) were further analyzed using post-hoc pairwise comparisons of the estimated marginal means (*emmeans* package in R). All post-hoc tests were two-sided and corrected for multiple comparisons using False discovery rate (FDR) correction for p-value adjustment. Comparisons were considered significant at adjusted-p < 0.05.

### 2.4 Analyses of resting-state networks

#### 2.4.1 Matching between spinal segmental and functional levels

The matching between anatomically derived segmental levels (*i.e.*, segmental organization defined based on Frostell et al. (2016)) and data-driven functional levels (*i.e.*, spinal resting-state networks, extracted using ICA and ICAP analyses) was estimated using the percentage of voxels of each IC or iCAP (thresholded at Z > 1.6 and binarized) falling in each spinal segmental level. Maximum weight matching (Crouse, 2016) was employed to match components and segmental levels.

#### 2.4.2 Robustness and replicability of the spinal networks

To assess the robustness (*i.e.*, between methods) and replicability (*i.e.*, between datasets) of the spinal resting-state networks, components maps were binarized (Z > 1.6) and Dice coefficients were computed to measure their spatial similarity. Maximum weight matching (Crouse, 2016) was employed to match components across methods and datasets. For the sake of completeness, Dice coefficients were also reported for Z > 2 and Z > 2.3, as well as for a threshold-free similarity measure (*i.e.*, 1 - cosine distance).

#### 2.4.3 Temporal stability of the spinal networks

To probe the temporal stability of the spinal networks, we evaluated whether networks identified from shorter time intervals of data remained consistent with those derived from full-length runs. To this end, we divided each participant’s functional run into two equal parts for the “mtl” dataset and four equal parts for the “gva” dataset, resulting in splits of approximately 3.5 min (132 and 90 volumes long for “mtl” and “gva”, respectively). We performed the ICA and iCAP procedures independently for each split, with K set to 9 for “mtl” and 5 for “gva.” We then computed Dice coefficients to evaluate the similarity between the spatial maps obtained from each split and those obtained from the entire run.

#### 2.4.4 Stability of individual-specific spinal networks

To evaluate the efficacy of ICA and iCAP pipelines in identifying individual-specific spinal functional levels, we conducted both analyses in each participant. The number of components (*i.e.*, K = 5 for “gva” participants and K = 9 for “mtl” participants) was selected to align with the expected segmental organization. To assess this matching, we computed the percentage of voxels of each IC or iCAP (thresholded at Z > 1.6 and binarized) falling in each anatomically derived spinal segmental level (Frostell et al., 2016). These overlap values were statistically tested for each dataset, between methods using two-sided paired-t-tests. Cohen’s d coefficients (d) were calculated to quantify the effect size of these tests (*t_to_d* function in the R package “effectsize”). To visualize the overall distribution of participant-specific components, heatmaps were generated by sorting ICs and ICAPs based on the center-of-mass of their largest clusters and summing the binarized components across participants.

To visually assess the correspondence between personalized components and individual neuroanatomy, we utilized the T2*w (for “mtl”) and T2w (for “gva”) anatomical images of each participant. Although these images were not specifically acquired for this purpose, we attempted to identify the approximate location of nerve roots. Both modalities were used to mark roots in the axial plane. However, due to the large slice thickness in the “mtl” T2*w images (5 mm), we exclusively used T2w images from the “gva” to isolate nerve roots in the coronal plane. To this end, we projected nerve roots and components along the dorso-ventral axis to enhance the visualization of their respective rostro-caudal positions (minimal intensity projection for the nerve roots and maximal intensity projection for the components).

## 3 Results

Hereafter, *anatomically derived levels* refer to the spinal segmental levels as defined in Frostell et al. (2016), while *functional levels* correspond to the data-driven levels extracted using ICA or iCAP.

### 3.1 Number of spinal levels and components

To achieve our main goal of functionally identifying the organization of the spinal cord, specifically its functional levels, we first determined the optimal number of independent components (ICs) or innovation-driven co-activation patterns (iCAPs) to extract (referred to as K). This determination was based on a combination of anatomical priors and stability measures.

Given that the neuron pools constituting functional levels give rise to the pairs of nerves forming segmental levels (Fig. 1a), we initially hypothesized that K should be matched with the expected number of segmental levels covered in each dataset, so as to delineate one functional component per level. To achieve this, we first assessed the spinal coverage of the masks used in the “mtl” and “gva” analyses (Fig. 1b) and determined the corresponding number of segmental levels based on anatomical knowledge (Frostell et al., 2016) (Fig. 1c). Our examination suggested a coverage of nine spinal segmental levels for the “mtl” dataset (C1 to T1 spinal segmental levels, corresponding to vertebral levels C1* to C7*), and five for the “gva” dataset (C5 to T1 spinal segmental levels, corresponding to vertebral levels C4* to C7*). These estimations were confirmed by visual identification of the nerve roots in axial views of the PAM50 template (Fig. 1d), corroborating the use of nine and five components to extract spinal functional levels in the “mtl” and “gva” datasets, respectively.

To further evaluate the validity of these choices as well as their functional stability, we extracted components in 100 random subsets (bootstrap across participants with replacement) of each dataset for different K values around these target values. Specifically, we tested their spatial similarity with components extracted using the full datasets in order to evaluate network stability across subsets (Fig. 1e). In the “mtl” dataset, both methods showed a peak value at K = 9 (0.75 [0.1] for ICA, reported as median Dice coefficient [interquartile range, IQR], and 0.92 [0.03] for iCAP), which had significantly higher subsampling stability than surrounding possibilities. For the “gva” dataset, a common peak value was observed at K = 5 for iCAP (0.9 [0.04]) and ICA (0.4 [0.14]), even though the latter also exhibited a higher peak value at K = 7 (0.51 [0.11]). Using a linear mixed model analysis, we confirmed that there were significant main effects of K values (in “gva”: χ2(4) = 94.8, p < 0.001, d = 0.44, for the iCAP method, and χ2(4) = 1706.4, p < 0.001, d = 1.85, for ICA; in “mtl” χ2(4) = 94.7, p < 0.001, d = 0.37 for iCAP, and χ2(4) = 446.4, p < 0.001, d = 0.94, for ICA). Post-hoc two-sided paired-t-tests (FDR-corrected) were used to highlight the statistical differences between the similarity values of the target K and surrounding K values (see details in Tables S1 and S2). For the sake of completeness, IC maps obtained for the observed stability peak at K = 7 are presented in Figure S2.

### 3.2 ICA and iCAP can robustly detect group spinal functional levels

**Fig. 2.**
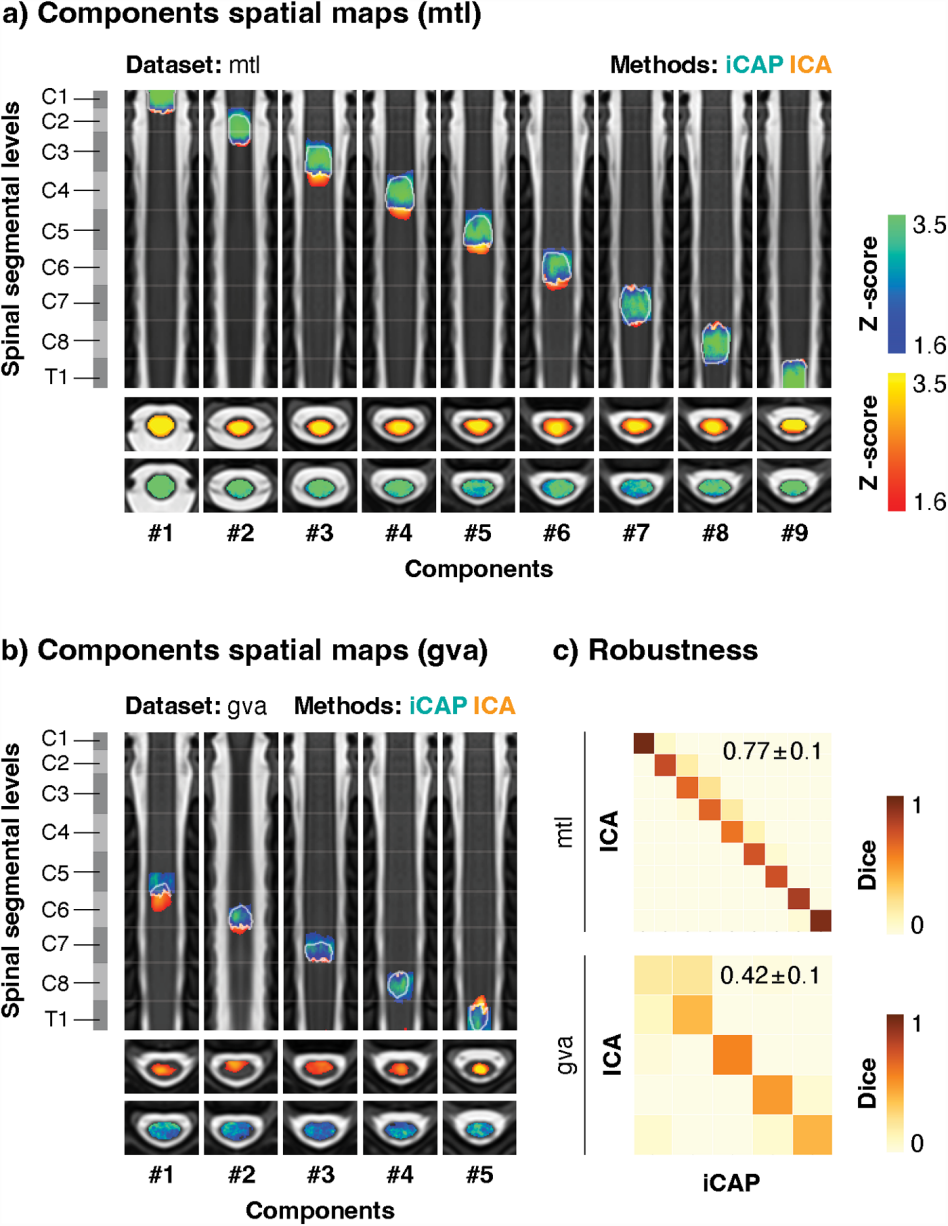
Uncovering spinal functional levels across methods. ICs (in yellow) and iCAPs (in blue) were extracted in the “mtl” (a) and the “gva” (b) datasets. Component maps (nine for “mtl” and five for “gva”) obtained with the two approaches are displayed on the PAM50-T2w template and presented in rostro-caudal order. A white outline indicates the overlap between two components. Coronal and axial views are presented. Spinal segmental levels derived from anatomical tables (Frostell et al., 2016) are displayed on the left for reference. Segment borders are represented on coronal views of the component maps by white lines of reduced opacity. The number of each component is provided below each map. (c) The robustness (*i.e.,* similarity across methods) of the spinal networks was evaluated using Dice coefficients between the binarized (Z > 1.6) ICs and iCAPs maps (see Table S5 for other similarity measures) for the “mtl” (top panel) and “gva” (bottom panel) datasets. The mean ± standard deviation Dice coefficient across matched components (*i.e.*, diagonal) is reported in the top right corner of each similarity matrix. IC: Independent components, ICA: Independent component analysis, iCAP: innovation-driven co-activation pattern analysis, SD: standard deviation.

Our investigation into the ability of data-driven approaches to reveal the functional architecture of the spinal cord began with an assessment of spinal organization at the group level in the “mtl” dataset, which encompasses the entire cervical spinal cord (C1 to T1). We first evaluated whether data-driven functional levels (derived using ICA or iCAP approaches) were in line with anatomical segmental levels (Frostell et al., 2016). Consistent with our hypothesis, extracting nine components using both ICA and iCAP methods revealed nine rostro-caudally segregated resting-state networks, seemingly corresponding to the spinal segmental levels covered in the dataset (Fig. 2a). To validate their accuracy, we assessed the spatial agreement of these components with the anatomically derived spinal segmental levels (Frostell et al., 2016). A substantial majority of component voxels—86.8 ± 16.2% (mean over components ± SD) for ICA and 92.5 ± 6.1 % for iCAP—matched segmental levels, confirming their alignment (see Table S3 for details). In addition, we observed a remarkable level of robustness across methods, evidenced by the high degree of spatial overlap between the functional segments identified using ICA and iCAP. This was confirmed by the strong diagonal pattern observed in the Dice coefficient matrix between the corresponding components maps (mean Dice ± SD across components = 0.77 ± 0.1, Figure 2c, see Table S5 for all similarity measures). Furthermore, the rostro-caudal positions of the component’s center of mass was also highly stable across methods (mean absolute distance between ICs and iCAPs z-axis center of mass ± SD, 2.44 ± 1.8 mm, more details in Table S3). For the sake of completeness, the same analyses were also conducted in the “gva” dataset (Fig. 2b, Table S4), highlighting that the segmental organization could be captured with overall robustness, although with a reduced agreement between methods (mean Dice ± SD across components = 0.42 ± 0.1, Figure 2c, see Table S5 for all similarity measures). For both datasets and methods, Figure S1e highlights the positions of components relative to tSNR variations, confirming their alignment with segmental levels rather than with periodic signal variations linked to intervertebral disks.

### 3.3 Spinal functional levels are replicable across datasets

**Fig. 3.**
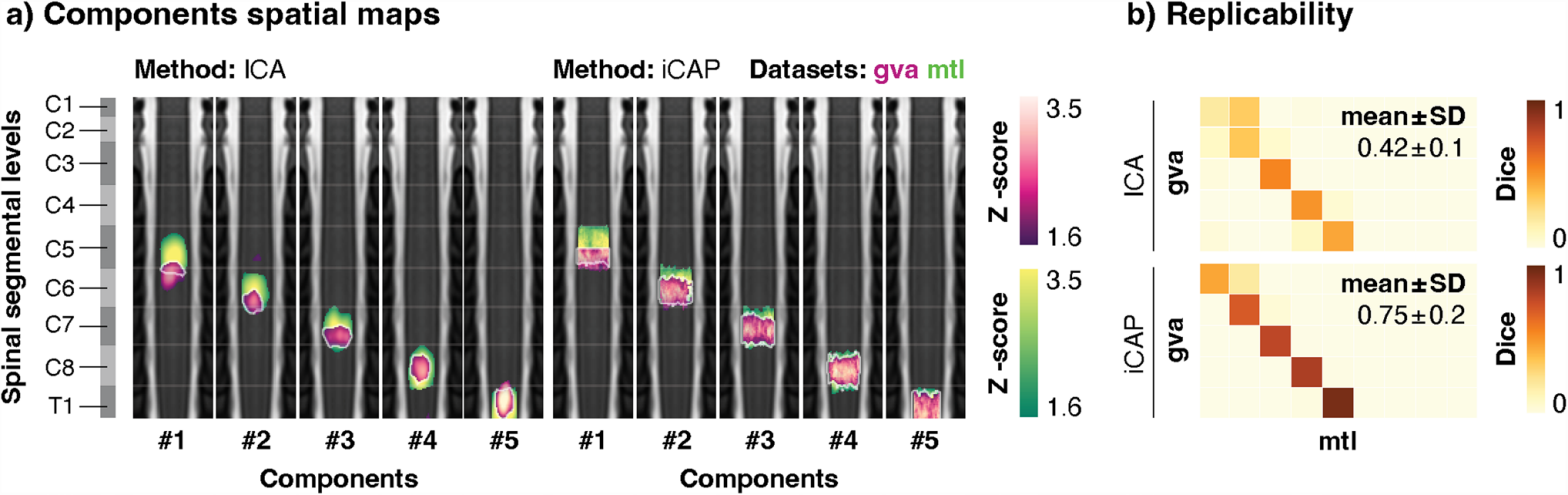
Uncovering spinal functional levels across datasets. (a) The components extracted from the “gva” dataset (in pink) were compared to the five more caudal components of the “mtl” one (in green). Component maps, presented in rostro-caudal order, are displayed on coronal views of the PAM50-T2w template. A white outline indicates the overlap between two components. Spinal segmental levels derived from anatomical tables (Frostell et al., 2016) are displayed on the left for reference. Segment borders are represented on coronal views of the component maps by white lines of reduced opacity. The number of each component is provided below each map. (b) The replicability (*i.e.,* similarity across datasets) of the spinal networks was evaluated using Dice coefficients between the maps obtained in the two datasets (“gva” *vs.* “mtl”). Results are presented for ICA (upper panel) and iCAP (lower panel). The mean ± standard deviation Dice coefficient (*i.e.*, diagonal) is reported in the top right corner of each similarity matrix. ICA: Independent component analysis, iCAP: innovation-driven co-activation pattern analysis, SD: standard deviation.

Next, we sought to extend our analysis beyond the “mtl” dataset and assess the replicability of our results (*i.e.,* similarity across “mtl” and “gva” datasets). To allow comparison, the five components extracted from the “gva” dataset were compared to the five more caudal components from the “mtl” dataset. As anticipated, these two sets of components largely aligned with spinal segmental levels C5 to T1, for both methods and datasets (Fig. 3a). While both methods enabled the retrieval of components underlying spinal functional levels, iCAPs appeared to be more replicable across datasets than their ICA counterparts (mean Dice coefficient across components ± SD = 0.42 ± 0.1 for ICA, and 0.75 ± 0.2 for iCAP) (Fig. 3b, see Table S5 for other similarity measures). In addition, we found that components extracted from the “gva” dataset exhibited a smaller extent than those of “mtl”, in particular for ICA (mean across components: “gva” = 2339 voxels *vs*. “mtl” = 5680 voxels, Tables S3 and S4), and to a lesser extent for iCAPs (“gva” = 5488 voxels *vs*. “mtl” = 7241 voxels). However, regardless of the components extent, the rostro-caudal positions of their centers of mass remained stable across datasets (mean ± SD absolute distance between “mtl” and “gva” z-axis center of mass for ICA: 3.3 ± 2.9 mm and iCAP: 2.4 ± 2.4 mm).

### 3.4 Spinal functional levels are temporally stable

**Fig. 4.**
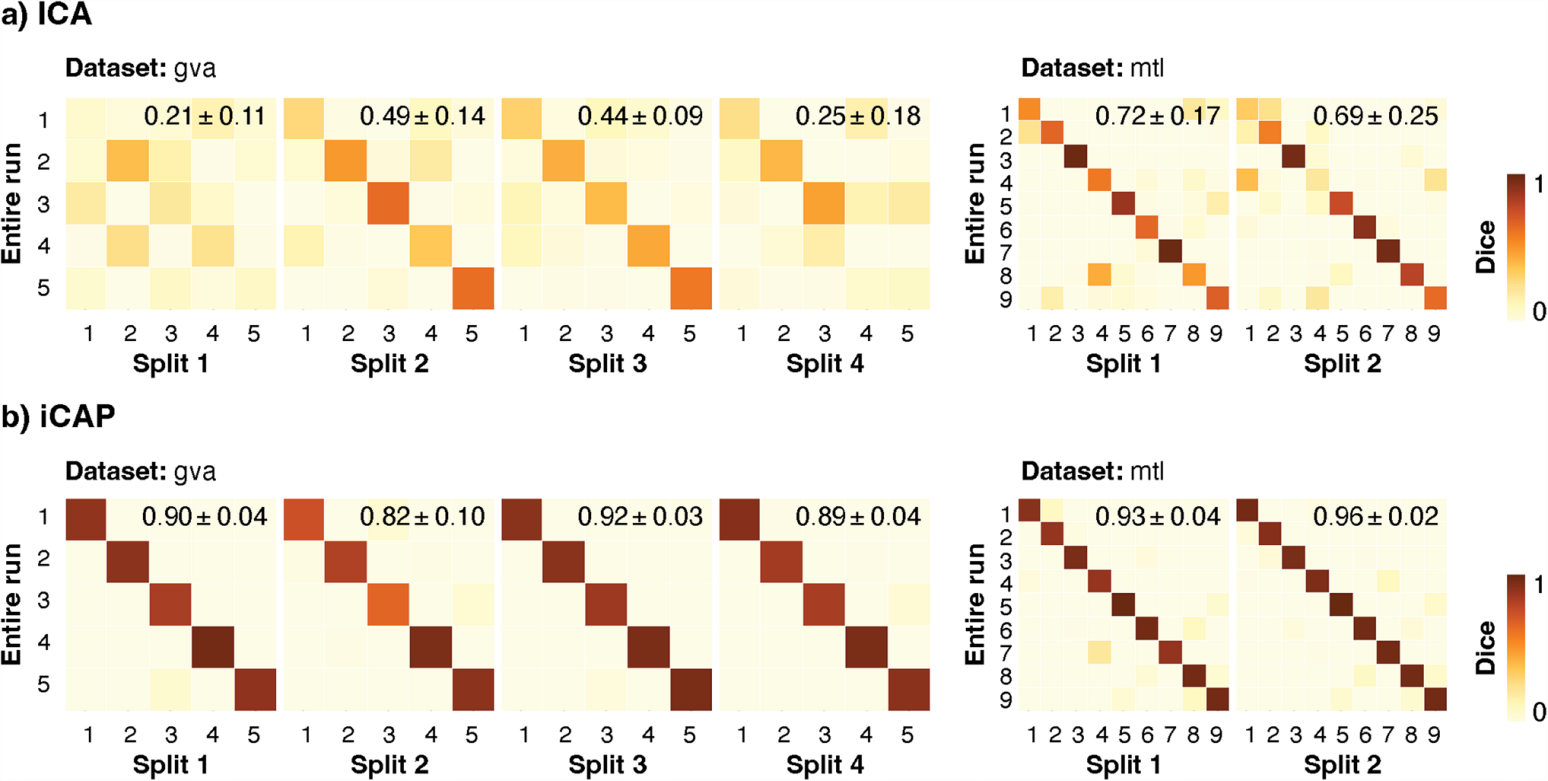
Uncovering spinal functional levels across time intervals. The temporal stability (*i.e.,* similarity across shorter time intervals) of the spinal networks was evaluated using Dice coefficients between components maps of each time split and those from the full-length run for ICA (a) and ICAP (b) methods in the two datasets (“gva” and “mtl”). Functional runs were divided into two and four equal parts for the “mtl” and the “gva” dataset, respectively. This resulted in splits lasting approximately 3.5 min for each dataset. The average diagonal Dice coefficient is reported in the top right corner of each similarity matrix (mean ± SD). ICA: Independent component analysis, iCAP: innovation-driven co-activation pattern analysis, SD: standard deviation.

We next assessed whether spinal functional levels could be reliably identified when the datasets were split in shorter time intervals. We observed that the impact of temporally dividing the runs varied depending on the method and dataset tested (Fig. 4). Specifically, ICA exhibited a lower ability to reliably identify spinal functional levels, with only two out of the four splits of the “gva” dataset showing fair performance (two fair Dice coefficients of 0.49 and 0.44 and two low Dice coefficients of 0.21 and 0.25, Fig. 4a). Performance was less strongly impacted for the “mtl” dataset, with ICs extracted in each split showing a high degree of spatial similarity (Dice coefficients of 0.72 and 0.69) with those of the entire run. In contrast, the performance of iCAP in identifying spinal functional levels was largely preserved in single splits (Fig. 4b), with Dice coefficients obtained between each split and the entire run ranging from 0.82 to 0.96 for both datasets.

### 3.5 Spinal functional organization is detectable at the individual level

To evaluate whether individual-specific spinal functional levels could be isolated in a data-driven manner, we extracted ICs and iCAPs for each participant from the two datasets. Notably, we observed limitations with ICA in identifying distinct resting-state networks at the individual level. While some components exhibited a clear segregation along the rostro-caudal axis (Fig. S3a), the majority of maps displayed a speckled pattern with small activations spanning multiple segments (Fig. S3b). Comparing these participant-level maps with anatomically derived segmental levels revealed a limited percentage of voxels aligning with the overall neuroanatomy (50.3 ± 6.7% for “mtl” and 51.7 ± 9% for “gva”, mean over participants ± SD). Likewise, the ICA-generated maps exhibited broad spatial distribution and demonstrated only a partial rostro-caudal organization (Fig. S3).

**Fig. 5.**
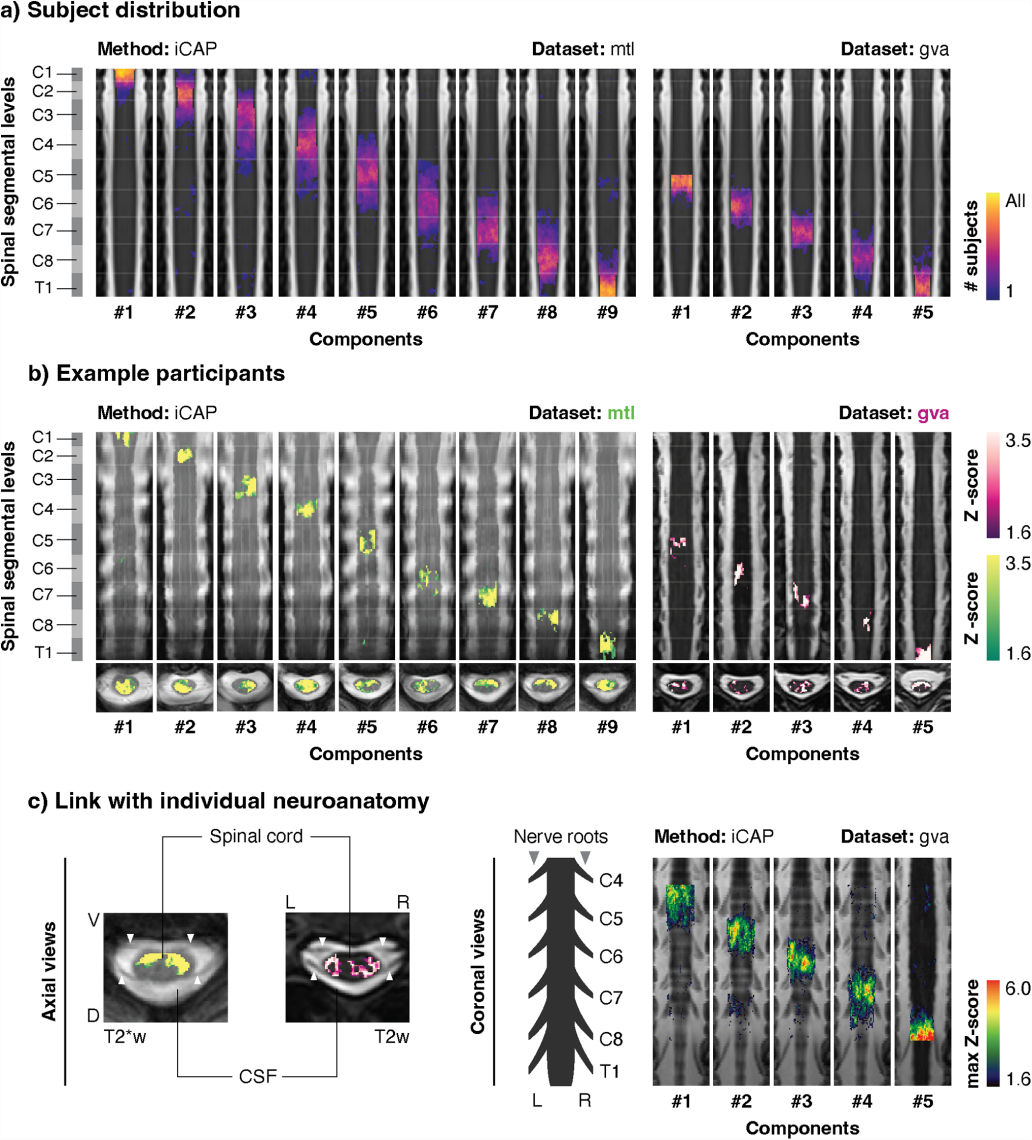
Uncovering spinal functional levels in individual participants. (a) Distribution maps of nine components (left, “mtl” dataset) and five components (right, “gva” dataset) extracted at the individual level using the iCAP approach. The heatmap represents the number of participants with a component corresponding to each spinal segmental level. Heatmaps are overlaid on coronal views of the PAM50-T2w template and presented in rostro-caudal order. Spinal segmental levels derived from anatomical tables (Frostell et al., 2016) are displayed on the left for reference. Segment borders are represented on component maps by white lines of reduced opacity. The number of each component is provided below each map. (b) Component maps extracted from one participant from “mtl” (green) and one participant from “gva” dataset (pink) using the iCAP approach. Functional maps are overlaid on coronal and axial views of the individual T2*w (“mtl”) and T2w (“gva”) anatomical images coregistered in the PAM50 space. (c) The left panel demonstrates the identification of nerve roots, highlighted by white arrows, on axial views in both T2*w (for “mtl”) and T2w (for “gva”) images. The right panel illustrates the detection of nerve roots in coronal views of the T2w images (*i.e.*, for “gva” only). Maps correspond to the maximum intensity projection of the maps shown for the “gva” participant panel (b), overlaid on a minimum intensity projection of the corresponding anatomical image to emphasize nerve roots. ICA: Independent component analysis, iCAP: innovation-driven co-activation pattern analysis.

In contrast, maps obtained using iCAP analysis had a narrow and well-organized distribution (Fig. 5a), similar to the arrangement of the group-level maps (Figs. 2 and 3). Accordingly, the correspondence with spinal segmental levels appeared to be more preserved in both the “mtl” (68.1 ± 6%) and “gva” (74.5 ± 8.7%) datasets compared to IC maps (iCAP vs ICA in “mtl” dataset: t(39) = 9.06, p < 0.001, d = 1.04; or “gva” dataset: t(35) = 7.97, p < 0.001, d = 0.85). The extraction of individualized iCAPs not only matched the overall topographic organization of spinal levels but also allowed for the capture of participant-specific organization of the spinal functional levels. We illustrate examples of these components extracted from two participants in each dataset in Figure 5b. While the anatomical images were not specifically acquired for this purpose, we attempted to leverage the T2*w (for “mtl”) and T2w (for “gva”) scans of each participant to gain a qualitative understanding of the matching with individual neuroanatomy (Fig. 5c). Axial views suggested that individual components maps fell in the vicinity of nerve roots (Fig. 5c, left panel). To better highlight this correspondence, maximal intensity projection maps were employed to emphasize the rostro-caudal organization of personalized spinal functional levels and their position relative to segmental levels (Fig. 5c, right panel; all participants are provided in Videos S1 and S2). This further confirmed that components were largely located in close proximity to the nerve roots

## 4 Discussion

Mapping the functional organization of the spinal cord *in vivo* in humans has been a long-standing challenge due to the lack of non-invasive and reliable techniques. In this study, we have taken a significant step towards addressing this limitation by demonstrating that spontaneous activity acquired using fMRI can be harnessed to map the topographic organization of spinal functional levels in a principled and data-driven manner, at both the group and individual scales. These findings have broad implications, notably for spinal cord fMRI processing, and as a first step towards future applications such as personalized diagnostics, and treatment monitoring.

Using two datasets (“mtl” and “gva”) with distinct acquisition protocols, as well as two different data-driven approaches (ICA and iCAP), we aimed to test the efficacy and reliability of these methods to uncover resting-state networks that would correspond to the spinal cord segmental levels. ICA is a powerful blind source separation technique that has been widely used to decompose brain fMRI datasets into a bilinear representation with spatially independent maps and associated time courses (Beckmann & Smith, 2004; Calhoun et al., 2001; Varoquaux et al., 2010). In contrast, iCAP is a more recent dynamic functional connectivity approach that identifies spatial patterns with similar functional dynamic behavior, characterized by simultaneous increases or decreases in activity (Karahanoğlu & Van De Ville, 2015). ICAP uses transient signals, also known as innovation signals, which are obtained as the derivative of the HRF-deconvolved fMRI time courses (Karahanoğlu et al., 2013). Then, clustering of the innovation volumes identifies consistent patterns of transient activity, termed iCAPs, which correspond to functional networks.

As a matter of fact, a handful of studies have explored spinal cord networks at varying levels of detail using ICA (Kong et al., 2014; Landelle et al., 2023) and iCAP (Kinany, Pirondini, Mattera, et al., 2022; Kinany et al., 2020). While they delineated networks exhibiting a distinct rostrocaudal organization (*e.g.*, alignment with vertebral levels (Kong et al., 2014)), these studies did not fully exploit their ability to specifically reveal the functional levels of the spinal cord architecture in a data-driven manner. Crucially, selecting the appropriate number of components to extract was a pivotal factor in this endeavor, as it can greatly impact the resulting networks. Given our aim of identifying spinal functional levels, we opted to set the number of components (K) to match the expected coverage in each dataset. In contrast to our earlier work (Kinany et al., 2020), where we defined coverage using a probabilistic atlas (De Leener et al., 2017), we refined our estimation by combining neuroanatomical knowledge (Frostell et al., 2016) and visual identification of nerve roots. The validity of these estimations was further underscored by the stability of components for the selected K values.

Using this procedure, we identified spinal resting-state networks aligned with neuroanatomy, as evidenced by their spatial convergence with segmental levels derived from anatomical tables (Frostell et al., 2016). Specifically, the extracted networks corresponded to spinal segments C1 to T1, and C5 to T1, for “mtl” and “gva,” respectively. These spinal segments play a crucial role in the transmission and integration of signals between the brain and the periphery, with the upper cervical segments (C1-C3) projecting to the face and neck, and the lower cervical segments as well as the first thoracic segments (C4-T1) to the upper limbs (Kendall et al., 2005). Importantly, unlike prior investigations that relied solely on assessing the rostro-caudal location of nerve rootlets (Cadotte et al., 2015) our data-driven approaches go beyond mere anatomical localization. By disentangling signals from distinct pools of neurons, they allow for the isolation of spinal levels with a functional emphasis, thus effectively reflecting the underlying spinal circuitry. The clear rostrocaudal segregation of the retrieved components suggests that this circuitry is predominantly intrinsic to individual spinal segments, rather than intersegmental. This observation aligns with earlier studies that reported either no significant correlations between resting-state components (Kong et al., 2014) or a decrease in functional connectivity as the distance between them increases (Kinany et al., 2020; Landelle et al., 2023; Wu et al., 2019). We anticipate that higher resolution imaging will offer better insight into the location of this functional intrinsic organization relative to the nerve root insertions. This holds great potential for future studies, enabling investigations into the modulation of these building blocks of the spinal cord’s functional architecture during tasks or following pathological conditions such as nerve compression or spinal cord injury.

To ensure the reproducibility of our findings, which is a major concern for scientific research and neuroimaging in particular (Nichols et al., 2017; Poldrack et al., 2017), we demonstrated the robustness (*i.e.,* across methods) and replicability (*i.e.,* across datasets) of the identified spinal cord networks. Specifically, our results outlined a large spatial agreement between components derived using ICA and iCAP, in both datasets. While networks derived using ICA exhibited a smaller spatial extent, likely owing to the spatial independence requirement of this approach, both methods could capture the rostro-caudal positions of spinal functional levels. It should be noted, however, that iCAP consistently outperformed ICA, especially in the “gva” dataset, which had a lower tSNR and temporal resolution. This underscores the potential of the dynamic iCAP approach, particularly when dealing with more challenging datasets.

Altogether, the generalizability of our results underscores the idea that an accurate, unbiased, and population-specific data-driven parcellation of the spinal cord topographic organization can be achieved. This is particularly relevant for region-of-interest based functional connectivity studies, where the choice of parcellation can significantly impact results (de Reus and van den Heuvel, 2013; Thirion et al., 2014). To date, such studies in the spinal cord (Eippert et al., 2017; Weber et al., 2018) have largely relied on probabilistic levels provided by the SCT (De Leener et al., 2017). Although atlases are useful in providing an estimate of the spinal cord organization, it is noteworthy that we observed a discrepancy between our data-driven spinal functional levels and the probabilistic segmental levels provided in the SCT, with the latter consistently shifted caudally, including a nearly one-level shift for T1 (see Fig. 1c). Several factors may contribute to these differences, including variations in normalization procedures and inter-participant variability. Indeed, SCT’s spinal segmental level locations are based on T2w anatomical scans of a separate group of 20 participants (Cadotte et al., 2015), which may not fully capture the variability in our populations. Moreover, nerve roots were only manually identified from C3 to C8, omitting spinal segmental levels C1 and C2, and extrapolating levels from T1 to T12, which potentially introduced inaccuracies. Therefore, our findings highlight the need for caution when interpreting results based on atlases and emphasize the importance of data-driven approaches in capturing the complex functional organization of the spinal cord. In line with the observed discrepancy, the atlas of segmental levels included in the SCT has been recently updated (v6.1 and later), aligning it with the spinal levels described in Frostell et al. (2016).

To further investigate the temporal stability of these data-driven parcellations and estimate the required data volume for reliable outcomes, we conducted additional analyses to probe the ability of ICA and iCAP methods to delineate spinal functional levels within shorter intervals of the data from the “mtl” and “gva” datasets (*i.e.*, splitting the datasets in distinct periods of time). While both methods could uncover spinal functional levels when provided with entire runs, iCAP outperformed ICA when the datasets were split into shorter intervals. This was particularly evident in the “gva” dataset, where ICA failed to identify levels in half of the data splits. Comparatively, networks extracted using iCAP in shorter periods remained highly stable for both datasets. We thus posit that the superiority of iCAPs may result from the unblurring effect obtained through HRF-deconvolution and the analysis of single volumes, making it less reliant on the amount of data compared to ICA. In sum, this outlines the potential of this method to retrieve spinal functional organization in settings where time or participant cooperation may be limited, such as clinical environments.

To advance spinal cord fMRI as a clinically valuable tool, it is crucial to move beyond group-level analyses and account for individual variations (O’Connor & Zeffiro, 2019). Indeed, although organized fluctuations have been documented utilizing slice-wise seed-based functional connectivity in individual participants (Barry et al., 2014), no study to date had explored their large-scale rostrocaudal arrangement. Our study marks a significant stride in this direction, as we present the first report of data-driven spinal resting-state networks in individual participants and demonstrate their ability to unveil personalized spinal functional levels. Of note, these individual-specific spinal levels exhibited noisier profiles than the group-level ones and could only be consistently observed using the iCAP framework. Again, we attribute this to the superiority of this approach in handling limited data, owing to the deconvolution-based denoising and the use of single frames. While this novel description of individualized spinal functional levels remains primarily qualitative, it lays a foundation for future research to build upon these auspicious results. Further investigations, possibly through multimodal anatomical-functional acquisitions, will provide a more comprehensive characterization of these personalized spinal resting-state networks, in order to unequivocally confirm their link with the underlying anatomy and probe their reliability. Additionally, utilizing highly sampled individuals, similar to previous studies in the brain (Gordon, Laumann, Gilmore, et al., 2017; Laumann et al., 2015), can shed light on their spatial properties and stability. From a methodological perspective, disentangling spinal functional levels in individual participants offers a promising avenue for improving inter-participant alignment and normalization procedures by using functional in place of traditional anatomical landmarks (Wang et al., 2015). Furthermore, segmental parcellations sensitive to individual variation may have broad implications for clinical practice, allowing for better surgical planning, for instance in the context of targeted spinal stimulation (Wagner et al., 2018), and facilitating the identification of functional biomarkers of disease (Wang et al., 2015).

In conclusion, our study demonstrates the potential of data-driven approaches as a powerful tool for identifying functional levels of the cervical spinal cord at both the group and individual levels. Although these results represent an important step in mapping the functional architecture of the spinal cord, it stands to reason that much remains to be explored. In particular, future studies could extend these investigations to a higher number of components. This extended analysis could yield multiple components per level, potentially allowing for the delineation of the dorsal and ventral horns of the gray matter. Thoroughly assessing the reliability of these finer parcellations will provide an unprecedented window into sensorimotor pathways. Additionally, a complete understanding of the spinal cord will require exploration of the organization of thoracic and lumbosacral regions, which can be challenging due to the smaller dimensions of spinal segmental levels in these regions (Frostell et al., 2016). Ultimately, the continued development of data-driven approaches to map the spinal cord will pave the way for a more comprehensive understanding of spinal function and dysfunction, with far-reaching implications for fundamental and clinical neuroscience.

## Supplementary Material

Supplementary Material

Kinany_Landelle_Video_S1

Kinany_Landelle_Video_S2

## Data Availability

The data for the “gva” dataset can be accessed on Mendeley Data with the identifier doi: 10.17632/n2k7zz2xyt.1. For the “mtl” dataset, the data are available upon reasonable request due to ongoing analyses and are not publicly accessible at this time. The code to conduct ICA analyses can be found in the Nilearn package, specifically the nilearn.decomposition.CanICA function. The code for conducting iCAP analysis can be accessed from the following source: https://c4science.ch/source/iCAPs/.
