# Supplementary material for: In vivo parcellation of the human spinal cord functional architecture
Source: Imaging Neurosci (Camb). 2024 Jan 11;2:imag-2-00059. doi: 10.1162/imag_a_00059 (PMC12235558; doi:10.1162/imag_a_00059)
Supplement: Supplementary Material [file imag_a_00059-supp.pdf]

Supplementary information

Signal quality check

We estimated the in-plane spinal motion by measuring the framewise displacement (FD) of the realignment parameters, which is an indicator that expresses instantaneous motion in X and Y direction. The averaged FD in the two directions was calculated for each dataset ('mtl': 0.1 [0.04] mm, 'gva': 0.1 [0.03] mm, reported as median FD coefficient [interquartile range, IQR]) and an analysis of variance was used to evaluate the dataset effect on this measure (Fig. S1). No significant dataset effect was observed ($F(1) = 1.43$, $p = 0.24$).

The temporal signal-to-noise ratio (tSNR) was performed to evaluate the quality of the signal over time during the acquisition of the functional images before preprocessing. For each voxel, the tSNR was obtained from the mean signal intensity across time divided by its standard deviation. The mean tSNR values were extracted in segmentation masks of the spinal cord ('mtl': 15.86 [2.65] and 'gva': 6.36 [0.9] mm, reported as median tSNR [interquartile range, IQR], Figure S1). Using an analysis of variance, we found that there were significant main effects of the datasets ($F(1) = 256.52$, $p < 0.001$).

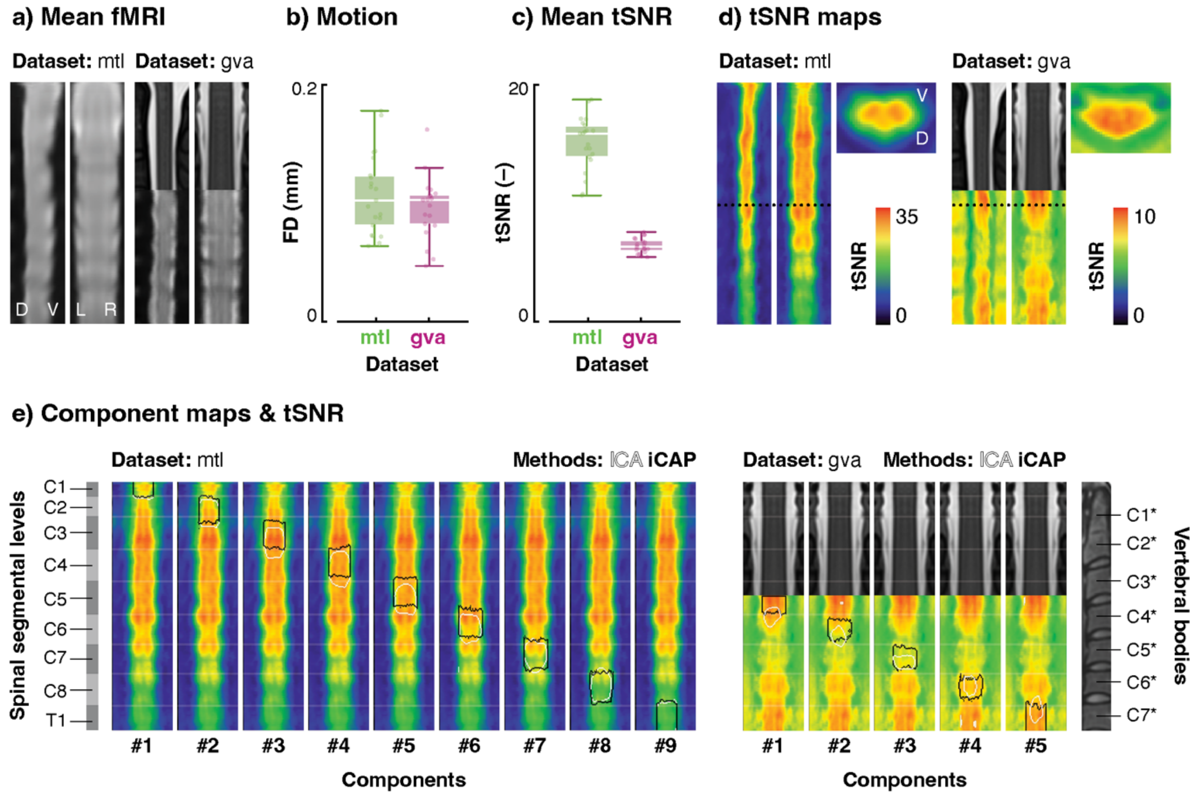

Figure S1 I Quality check. (a) Mean functional images after normalization, overlaid on the PAM50 template. Sagittal and coronal views are presented. **(b)** Boxplot of the framewise displacement (FD) across datasets ('mtl' in green and 'gva' in pink), with medians represented by the horizontal white line inside the box. Vertical lines denote the extreme values within a 1.5 interquartile range and dots represent values for each

individual. **(c)** Boxplot of the temporal signal noise ratio (tSNR, mean value in the spinal cord mask) to compare the global tSNR across datasets **(d)** Group average tSNR maps are overlaid on the PAM50-T2w image to depict the spatial variations of the tSNR within each dataset. Sagittal, coronal, and axial (position indicated by the dotted lines) views are presented. **(e)** Components maps (ICs outlined in white, iCAPs outlined in black) are overlaid on tSNR maps for 'mtl' (left panel) and 'gva' (right panel) datasets. Spinal segmental levels derived from anatomical tables (Frostell et al., 2016) are displayed on the left for reference. Segment borders are represented on coronal views of the component maps by white lines of reduced opacity. Vertebral bodies (PAM50 template) are provided on the right. D: dorsal, L: left, R: Right, V: ventral.

Number of spinal levels and components

Table S1 l Summary of the statistical results from post-hoc paired-tests (FDR-corrected) analysis in the 'gva' dataset.

Post-hoc	*a- ICA*				*b- iCAP*		
	df	*t-value*	*p-value*		*df*	*t-value*	*p-value*
**K5 *vs.* K3**	404	25.9	<0.0001		404	6.9	<0.0001
**K5 *vs.* K4**	404	20.4	<0.0001		404	9.3	<0.0001
**K5 *vs.* K6**	404	9.8	<0.0001		404	4.2	0.0001
**K5 *vs.* K7**	404	-9.6	<0.0001		404	6.3	<0.0001

iCAP: innovation-driven co-activation pattern analysis, ICA: Independent component analysis, K: number of ICs or ICAPs extracted, df: degree of freedom

Table S2 l Summary of the statistical results from post-hoc paired-tests (FDR-corrected) analysis in the 'mtl' dataset.

Post-hoc	*A- ICA*				*B- iCAP*		
	df	*t-value*	*p-value*		*df*	*t-value*	*p-value*
**K9 *vs.* K7**	404	17.9	<0.0001		404	7.8	<0.0001
**K9 *vs.* K8**	404	10.9	<0.0001		404	3.3	<0.0015
**K9 *vs.* K10**	404	6.5	<0.0001		404	3.5	0.0010
**K9 *vs.* K11**	404	16.8	<0.0001		404	5.9	<0.0001

K: number of ICs or ICAPs extracted, df: degree of freedom

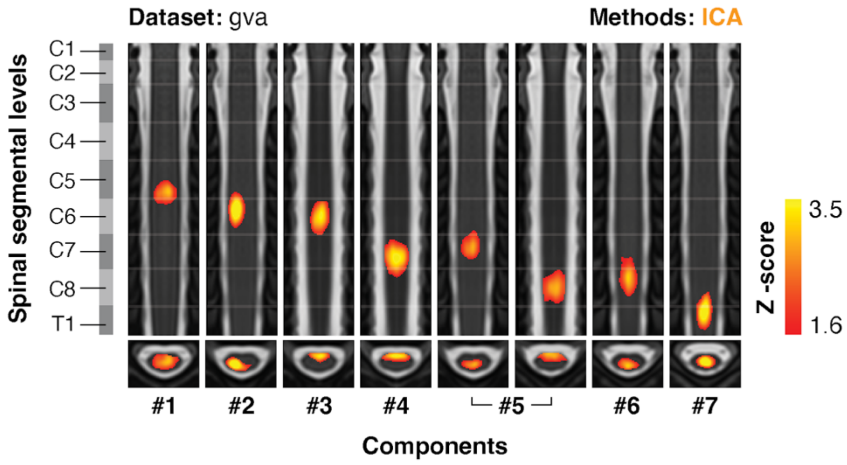

Figure S2 I ICs for K = 7 in the 'gva' dataset I While we selected K = 5 for the 'gva' dataset, based on a common stability peak observed for both ICA and iCAP at this granularity level, coupled with anatomical knowledge, we additionally extracted the 7 ICs corresponding to the observed stability peak for the ICA condition alone. These components, while aligning with the spinal cord's segmental organization, exhibited a tendency to divide into dorsal and ventral components or fragment into multiple clusters spanning different levels (see component #5). Component maps (in yellow) are displayed on coronal views of the PAM50-T2w template and presented based on the rostro-caudal position of their center-of-gravity. Spinal segmental levels derived from anatomical tables (Frostell et al., 2016) are displayed on the left for reference. Segment borders are represented on component maps by white lines of reduced opacity. The number of each component is provided below each map.

Spinal functional organization at the group-level

Table S3 I Spinal cord resting-state components extracted with ICA **(a)** or iCAP **(b)** approach in the 'mtl' dataset

Comp label	*a- ICA*							*b- iCAP*					
	vox	*x*	*y*	*z*	*z-score*	*% vox*		*vox*	*x*	*y*	*z*	*z-score*	*% vox*
**C1**	5680	30	27	287	4.4	99.4		5562	30	27	288	5.2	100
**C2**	5337	30	25	262	4.0	52.1		6389	30	25	266	6.0	84.1
**C3**	5286	30	25	233	4.1	68.9		7112	30	25	240	4.8	92.3
**C4**	6017	30	25	206	3.6	83.1		7366	30	26	213	4.6	98.4
**C5**	5770	30	26	175	4.0	97.4		7900	30	26	183	4.4	89.8
**C6**	6124	31	25	147	3.7	99.4		8319	30	26	153	4.3	84.9
**C7**	5708	31	26	120	3.8	93.4		8985	30	26	121	4.2	89.3
**C8**	5805	30	26	88	4.0	94.7		7754	30	26	88	4.5	93.4
**T1**	5396	30	26	69	4.3	93.7		5785	30	26	59	5.0	99.8

The components are labeled according to the corresponding spinal segmental level. vox represents their number of voxels. Coordinates of the component center of mass are denoted by x,y,z (in PAM50 space, voxel location for a resolution of 0.5mm). z-score refers to the maximum value of the component. % vox represents the percentage of voxels matching the corresponding spinal segmental level. iCAP: innovation-driven co-activation pattern analysis, ICA: Independent component analysis.

Table S4 I Spinal cord resting-state components extracted with ICA **(a)** or iCAP **(b)** method in the ‘gva’ dataset

**Comp label**	***a- ICA***						***b- iCAP***					
	vox	***x***	***y***	***z***	***z-score***	***% vox***	***vox***	***x***	***y***	***z***	***z-score***	***% vox***
**C5**	2425	31	25	159	3.0	29.5	5139	30	26	171	3.9	97.5
**C6**	2275	31	29	140	2.9	79.2	5572	31	26	146	3.7	100
**C7**	2671	31	27	114	2.5	93.3	5875	30	26	118	3.4	99.9
**C8**	2085	31	26	89	2.7	89.2	5537	30	26	87	3.9	99.9
**T1**	2241	30	25	66	3.9	59.1	5319	30	26	58	3.7	98.4

The components are labeled according to the corresponding spinal segmental level. vox represents their number of voxels. Coordinates of the component center of mass are denoted by x,y,z (in PAM50 space,voxel location for a resolution of 0.5mm). z-score refers to the maximum value of the component. % vox represents the percentage of voxels matching the corresponding spinal segmental level. iCAP: innovation-driven co-activation pattern analysis, ICA: Independent component analysis.

Table S5 I Component similarity using Dice coefficients with different Z thresholds (a) and Cosine similarity (b)

		**a- Dice**			b- Cosine
		Z > 1.6	Z > 2	Z > 2.3	threshold-free
Robustness	gva	0.42 ± 0.1	0.28 ± 0.1	0.16 ± 0.1	0.33 ± 0.2
	mtl	0.77 ± 0.1	0.69 ± 01	0.63 ± 0.2	0.64 ± 0.1
Replicability	ICA	0.42 ± 0.1	0.32 ± 0.1	0.19 ± 0.1	0.34 ± 0.1
	iCAP	0.75 ± 0.1	0.65 ± 0.2	0.51 ± 0.2	0.86 ± 0.2

Spinal functional organization at the individual level

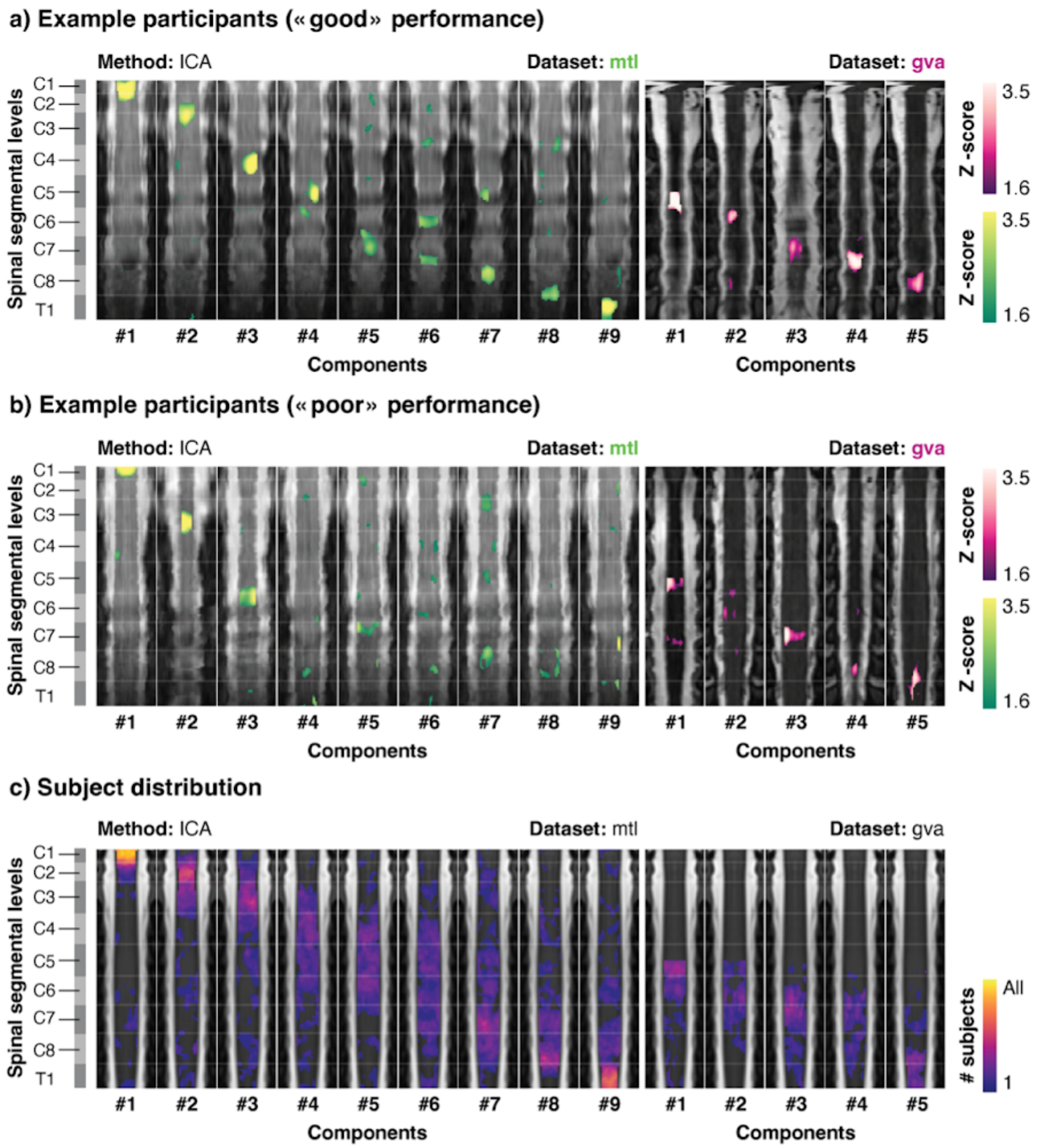

Figure S3 | Uncovering spinal levels in individual participants. (a-b) Component maps extracted from individual participants from ‘mtl’ (green) and participants from ‘gva’ dataset (pink) using the ICA approach, with “good” **(a)** or “poor” **(b)** performance. Functional maps are overlaid on coronal and axial views of the individual T2*w (‘mtl’) and T2w (‘gva’) anatomical images coregistered in the PAM50 space and displayed in rostro-caudal order. Spinal segmental levels derived from anatomical tables (Frostell et al., 2016) are displayed on the left for reference. Segment borders are represented on component maps by white lines of reduced opacity. The number of each component is provided below each map. **(c)** Distribution maps of 9 components (left, ‘mtl’ dataset) and 5 components (right, ‘gva’ dataset) extracted at individual level using the ICA approach. The heatmap represents the number of participants with a component corresponding to each spinal segmental level. Functional maps are overlaid on the PAM50-T2w image. ICA: Independent component analysis.
